# Non-adiabatic Quantum Dynamics of the Dissociative Charge Transfer He^+^+H_2_ → He+H+H^+^

**DOI:** 10.3389/fchem.2019.00249

**Published:** 2019-04-16

**Authors:** Dario De Fazio, Alfredo Aguado, Carlo Petrongolo

**Affiliations:** ^1^Consiglio Nazionale delle Ricerche, Istituto di Struttura della Materia, Rome, Italy; ^2^Departamento de Química Física Aplicada, Facultad de Ciencias, Universidad Autónoma de Madrid, Madrid, Spain; ^3^Consiglio Nazionale delle Ricerche, Istituto per i Processi Chimico Fisici, Pisa, Italy

**Keywords:** He^+^+H_2_, wavepacket, conical intersection, non-adiabatic, quantum, dynamics

## Abstract

We present the non-adiabatic, conical-intersection quantum dynamics of the title collision where reactants and products are in the ground electronic states. Initial-state-resolved reaction probabilities, total integral cross sections, and rate constants of two H_2_ vibrational states, *v*_0_ = 0 and 1, in the ground rotational state (*j*_0_ = 0) are obtained at collision energies *E*_coll_ ≤ 3 eV. We employ the lowest two excited diabatic electronic states of ${\text{HeH}}_{2}^{+}$ and their electronic coupling, a coupled-channel time-dependent real wavepacket method, and a flux analysis. Both probabilities and cross sections present a few groups of resonances at low *E*_coll_, whose amplitudes decrease with the energy, due to an ion-induced dipole interaction in the entrance channel. At higher *E*_coll_, reaction probabilities and cross sections increase monotonically up to 3 eV, remaining however quite small. When H_2_ is in the *v*_0_ = 1 state, the reactivity increases by ~2 orders of magnitude at the lowest energies and by ~1 order at the highest ones. Initial-state resolved rate constants at room temperature are equal to 1.74 × 10^−14^ and to 1.98 × 10^−12^ cm^3^s^−1^ at *v*_0_ = 0 and 1, respectively. Test calculations for H_2_ at *j*_0_ = 1 show that the probabilities can be enhanced by a factor of ~1/3, that is *ortho-*H_2_ seems ~4 times more reactive than *para-*H_2_.

## Introduction

Atomic Hydrogen and Helium are the dominant chemical species of the early Universe (Galli and Palla, 2013) and of the interstellar medium, and are easily ionized by cosmic rays. Therefore, these atoms and their ions are the astrochemical fundamental reactants (Lepp et al., 2002), together with ubiquitous photons, giving first simple diatoms as H_2_, ${\text{H}}_{2}^{+}$ (Stancil et al., 1993), and HeH^+^ (Zygelman et al., 1998) and then atom+diatom bimolecular collisions as He+${\text{H}}_{2}^{+}$ (De Fazio et al., 2012), H+HeH^+^ (De Fazio, 2014; Gamallo et al., 2015), and He^+^+H_2_.

When all chemical species are in the ground electronic states, the He+${\text{H}}_{2}^{+}$ reaction is endothermic and rather slow, but the other two are exothermic by about ~0.7 and 6.2 eV, respectively, as many ion+neutral astrochemical reactions (Herbst and Klemperer, 1973). H+HeH^+^ is barrierless and gives quickly the He+${\text{H}}_{2}^{+}$ products, but the collision-induced dissociative charge transfer (DCT) He^+^(^2^*S*)+H_2_(*X*^1^Σ${}_{g}^{+}$) → He(^1^*S*)+H(^2^*S*)+H^+^ is very slow at low collision energy *E*_coll_ and at room temperature, if H_2_ is in the ground vibro-rotational state. In fact, H+HeH^+^↔He^+^+H_2_ occurs on the ground potential energy surface (PES) $\tilde{X}$^2^*A*' of ${\text{HeH}}_{2}^{+}$, which is well-separated from the excited electronic species, but the low-*E*_coll_ DCT involves the first two excited adiabatic electronic states Ã^2^*A*' and $\tilde{B}$^2^*A*' of ${\text{HeH}}_{2}^{+}$, which differ by a two-electron excitation and are coupled by a *C*_2*v*_ conical intersection (CI) (Preston et al., 1978; McLaughlin and Thompson, 1979). In the latter case the non-adiabatic coupling is weak and the lower, dissociative cone of the intersection seam gives rise to an adiabatic barrier with diabatic character, that is the DCT tends to follow the diabatic PESs without changing the electronic configuration. This strongly inhibits the reactivity that is associated with the tunneling through the barrier (Preston et al., 1978).

**Figure 1:**
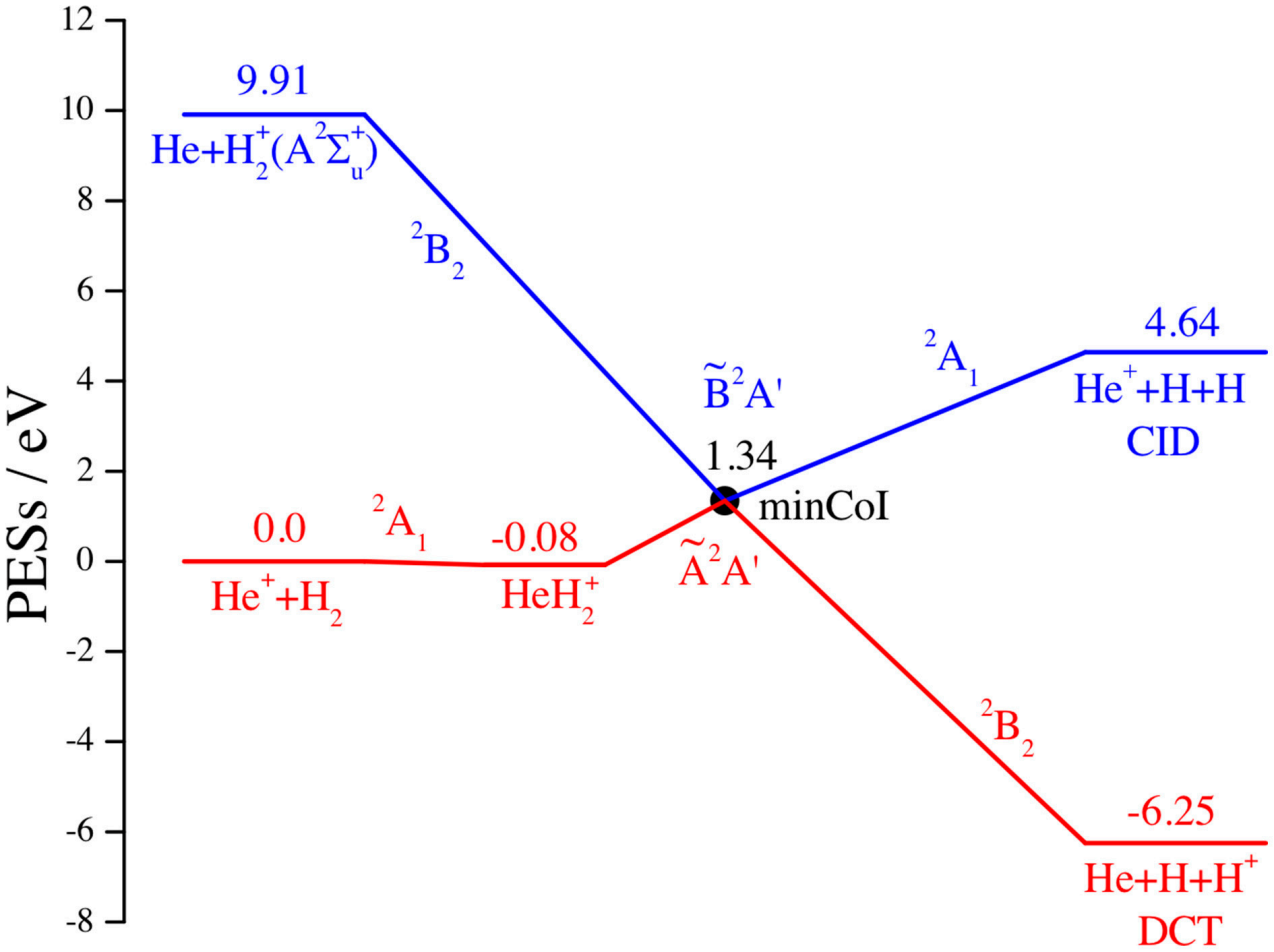
Dissociative charge transfer (DCT) and collision induced dissociation (CID) of He^+^+H_2_. Schematic correlation diagram in eV of the adiabatic Ã^2^*A*′ (red) and $\tilde{B}$^2^*A*′ (blue) and of the diabatic (1)^2^*A*_1_ and (2)^2^*B*_2_ PESs. All reactants and products are in the ground electronic state, save ${\text{H}}_{2}^{+}$(*A*^2^Σ${}_{u}^{+}$, unbound).

This is schematically shown in the correlation diagram of Figure 1 for the Ã^2^*A*' and $\tilde{B}$^2^*A*' adiabatic and (1)^2^*A*_1_ and (2)^2^*B*_2_ diabatic PESs *V* of ${\text{HeH}}_{2}^{+}$, where all chemical species are in the ground electronic states, save ${\text{H}}_{2}^{+}$(*A*^2^Σ${}_{u}^{+}$) that is unbound, and the energy is referred to the reactant minimum. This diagram is obtained from the *ab initio* Multi-Reference Configuration-Interaction results of McLaughlin and Thompson (1979), changing the label of the reactant diabatic state ^2^*A*_1_ from (5) to (1), and from the analytical fits of the associated diabatic PESs *V*_11_ and *V*_22_ by Aguado et al. (1993). In the scheme of Figure 1 we omit the ground adiabatic PES, well below the excited PESs and with too small non-adiabatic couplings. However, all three adiabatic PESs are strongly coupled by intense laser pulses (Szidarovszky and Yamanouchi, 2016) when the ground PES becomes populated (Schauer et al., 1989). A more complete description of the reaction dynamics in presence of electric and magnetic fields is out of the scope of the present article. Note in the figure the small ion-induced dipole minimum in the *C*_2v_ entrance channel at *R*(He-H_2_) = 4.45 *a*_0_, *r*(H-H) = 1.42 *a*_0_, and *V* = −0.08 eV and the CI *C*_2*v*_ minimum at *R* = 4.89 *a*_0_, *r* = 2.18 *a*_0_, and *V* = 1.34 eV, owing to the intersection (Preston et al., 1978) between the H_2_(*X*^1^Σ${}_{g}^{+}$) and ${\text{H}}_{2}^{+}$(*A*^2^Σ${}_{u}^{+}$) curves at *r* = 2.19 *a*_0_ and *V* = 1.28 eV.

Experimental studies on the He^+^+H_2_ → He+H+H^+^ DCT dynamics date back to 1955 (Stedeford and Hasted, 1955) and 1961 (Giese and Maier, 1961), when the integral cross sections (ICSs) were measured at *E*_coll_ = 4 eV and found <0.05 Å^2^. ICSs as functions of *E*_coll_ were then measured in many works up to 1996 (Dhuicq et al., 1996), finding values from 0.01 up to ~2 Å^2^, for *E*_coll_ between 3 and 100 eV, by considering H_2_ in the ground vibro-rotational state and all ground and excited open states of H. Below 3 eV, the H product is in the ground 1*s* state and the ICSs are so small and so difficult to measure that experimental values present large discrepancies (Reinig et al., 1992). However, the ICSs increase by one/two orders of magnitude if H_2_ is excited by one vibrational quantum (Preston et al., 1978) or *E*_coll_ grows up to ~100 eV. Differential cross sections were also measured (Dhuicq et al., 1998) at *E*_coll_ > 9 eV, that is for the formation of the H^*^ excited product. Accordingly, small rate constants were observed (Johnsen et al., 1980), with values of (1.5 ± 0.15) × 10^−13^ and (1.1 ± 0.1) × 10^−13^cm^3^s^−1^ at 78 and 330 K, respectively, that is nearly four order of magnitude lower than the Langevin (Gioumousis and Stevenson, 1958) estimates for ion+neutral barrierless and exothermic reactions.

A few works have also theoretically investigated the dynamics of the DCT collision since 1994, when Aguillon (1994) employed a semiclassical coupled wavepacket (WP) method and the analytical diabatic PESs of Aguado et al. (1993) for computing ICSs for ground and excited vibrational states *v*_0_ of H_2_, up to *v*_0_ = 4 and in the *E*_coll_ range from 2 to 10 eV. He found total-ICS values from ~0.001 Å^2^ (*v*_0_ = 0, *E*_coll_ = 2 eV) to ~1 Å^2^ (*v*_0_ = 3, *E*_coll_ = 10 eV), in agreement with the most recent measurements (Dhuicq et al., 1996). In a subsequent work (Aguillon, 1998), an improved version of this semiclassical method was used for obtaining new ICS values and differential cross sections. Approximated theoretical models were also employed (Dhuicq et al., 1996) for explaining observed cross sections above 9 eV, when excited H^*^(*n* = 2) was produced. Finally, quantum infinite-order-sudden cross sections were calculated (Martínez et al., 2000) for He^+^+H_2_ → He+H^*^(*n* ≥ 2)+H^+^ at *E*_coll_ ≥ 10 eV, using the accurate diabatic representation of Aguado et al. (1993) and six more approximated diabatic electronic states (Sidis, 1996).

As far as we know, no further studies of the title reaction have been published and its collision dynamics below ~2 eV is unknown. In particular, only semiclassical or approximated quantum theoretical studies were carried out, although both conical intersection and barrier tunneling are purely quantum effects. We thus here report a rigorous time-dependent quantum study of the DCT He^+^+H_2_(*v*_0_ = 0,1) → He+H+H^+^ with all species in the ground electronic state, at thermal and hyperthermal collision energy up to 3 eV, using the diabatic PESs of Aguado et al. (1993) and WP and flux formalisms. In section Theory and Calculations we present the theoretical method and its numerical details. Section Collision Results reports initial-state-resolved total reaction probabilities, ICSs, and thermal rate constants. Finally, we present our conclusions in section Conclusions.

## Theory and Calculations

### Potential Energy Surfaces and Non-adiabatic Coupling

As we said in the Introduction, the ${\text{HeH}}_{2}^{+}$ adiabatic and diabatic electronic states relevant in the present work were obtained *ab initio* in McLaughlin and Thompson (1979), fitted analytically in Aguado et al. (1993), and they are schematically plotted in Figure 1. We label the adiabatic species and PESs by Ã^2^*A*' and $\tilde{B}$^2^*A*' and by *V*_*A*_ and *V*_*B*_, respectively. As already discussed (Aguado et al., 1993), these states belong to the fully symmetric irreducible representation for linear (*C*_∞*v*_) and non-symmetric (*C*_*S*_) geometries, while Figure 1 shows that they transform as *A*_1_/*B*_2_ and *B*_2_/*A*_1_ for perpendicular geometries (*C*_2v_), before/after the CI, respectively, which rules the title reaction. On the other hand, we label the associated diabatic electronic states and PESs by (1)*A*_1_ and (2)*B*_2_ and by *V*_11_, *V*_22_, and *V*_12_, respectively, where the third surface describes the CI non-adiabatic coupling in the diabatic representation.

In order to simultaneously describe these electronic species and take into account the CI, a fit of the diabatic PESs and coupling *V*_11_, *V*_22_, and *V*_12_ was made in Aguado et al. (1993). The description of the adiabatic states is obtained as the eigenvalues *V*_*A*_ and *V*_*B*_ of a 2 × 2 matrix $\;\left(\begin{array}{cc} V_{11} & V_{12}\\ V_{12} & V_{22} \end{array}\right)\;$, in which the interaction term *V*_12_ must have the correct symmetry, being anti-symmetric with respect to the permutation of the H atoms and thus vanishing identically for equal He–H distances. This coupling term *V*_12_ was fitted in Aguado et al. (1993) to reproduce the CI between the Ã^2^*A*' and $\tilde{B}$^2^*A*' adiabatic states.

The diabatic surfaces where fitted in Aguado et al. (1993) using the Aguado-Paniagua functional form (Aguado and Paniagua, 1992; Aguado et al., 1998) that expands the energy as a multidimensional permutationally invariant polynomial (Aguado et al., 1994, 2001) in Rydberg type variables (Rydberg, 1931) of the form ρ_AB_ = *R*_AB_exp(−β_AB_*R*_AB_), where A and B are two nuclei and *R*_AB_ is their distance. For the interaction term, a simple expansion in Rydberg functions,

(1) $$\begin{array}{l} V_{12}\left(R_{\text{HeH}},R_{\text{He}{\text{H}}^{\prime }},R_{\text{H}{\text{H}}^{\prime }}\right)=C_{12}\rho_{\text{H}{\text{H}}^{\prime }}\left(\rho_{\text{HeH}}-\rho_{\text{He}{\text{H}}^{\prime }}\right), \end{array}$$

fulfills the anti-symmetric requirement.

**Figure 2:**
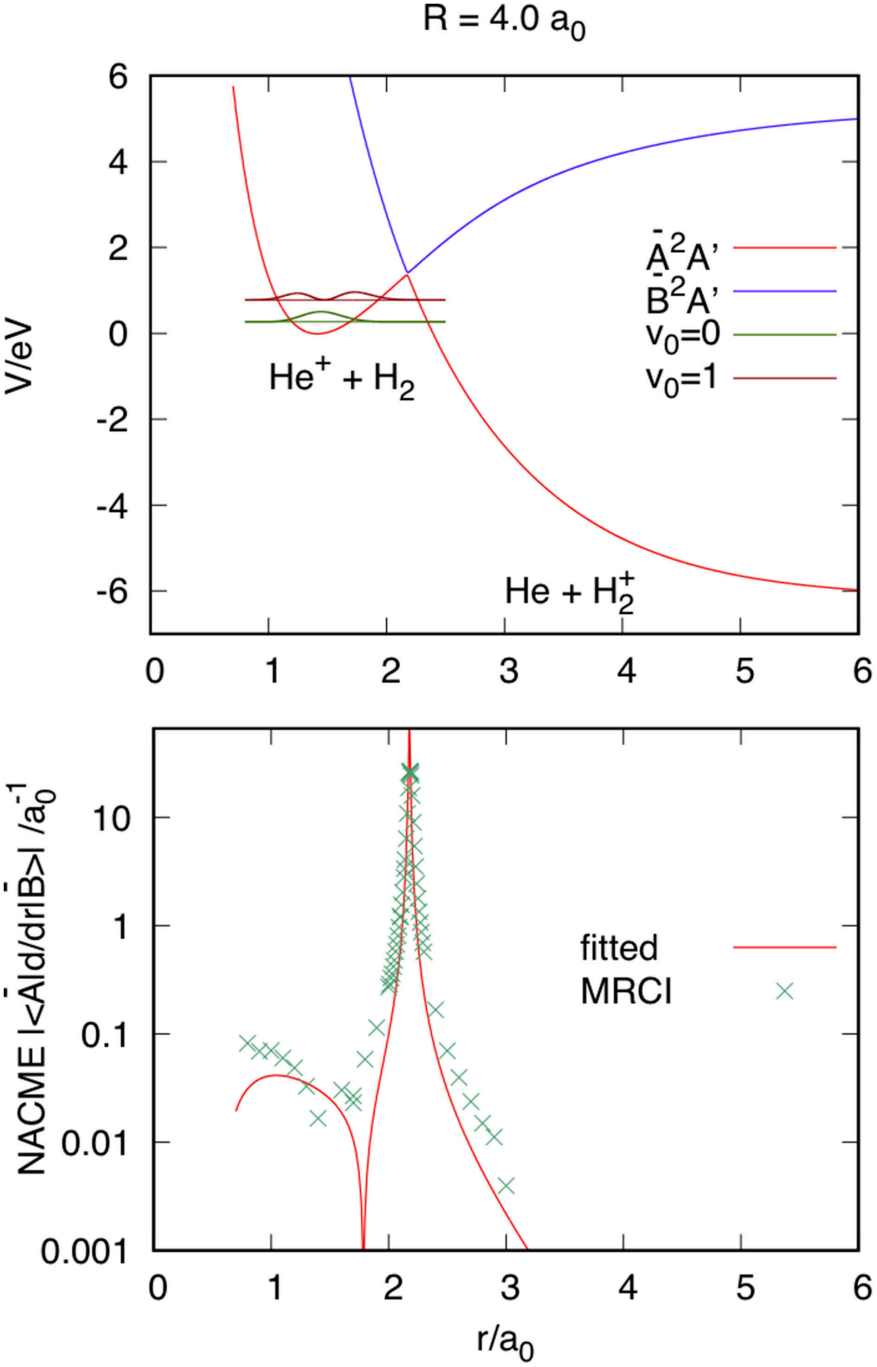
Top panel: Ã^2^*A*' and $\tilde{B}$^2^*A*' adiabatic PESs at reactant Jacobi coordinates *R* = 4 *a*_0_, *r*, and γ = 0. The vibrational *v*_0_ = 0 and 1 probability density is plotted at the corresponding vibrational energy. Bottom panel: Comparison of the absolute values of the fitted and *ab initio* Multi-Reference Configuration-Interaction (MRCI) NACME.

The DCT is produced through the CI, as shown in Figure 2 using nuclear Jacobi coordinates *R, r* = *R*${}_{\text{H}{\text{H}}^{\text{′}}}$, and γ , at *R* = 4 *a*_0_ and γ = 0. The top panel shows the adiabatic Ã^2^*A*' and $\tilde{B}$^2^*A*' PESs of ${\text{HeH}}_{2}^{+}$ in the reactant channel, obtained from the diabatic *V*_ij_ surfaces, where Ã^2^*A*' correlates with He^+^(^2^*S*)+H_2_(*X*^1^Σ${}_{g}^{+}$) for *r* < 2 *a*_0_ and with He(^1^*S*)+${\text{H}}_{2}^{+}$($A^{2}\Sigma_{u}^{+}$, unbound) for *r* > 2 *a*_0_. To analyze the accuracy of the fit, we compare the analytical non-adiabatic coupling matrix elements (NACMEs) in the adiabatic representation, obtained from the fitted diabatic energies and coupling, with the *ab initio* results calculated using the MOLPRO program package (Werner et al., 2018). As done previously for ${\text{H}}_{4}^{+}$ and ${\text{H}}_{5}^{+}$ in Sanz-Sanz et al. (2015), the analytical NACMEs can be calculated from the generalized Hellmann-Feynman theorem,

(2) $$\langle {\tilde{A}}^{2}A^{\prime }|\frac{\partial }{\partial R_{\text{AB}}}|{\tilde{B}}^{2}A^{\prime }\rangle =\frac{1}{V_{B}-V_{A}}\langle {\tilde{A}}^{2}A^{\prime }|\frac{\partial {\hat{H}}^{el}}{\partial R_{\text{AB}}}|{\tilde{B}}^{2}A^{\prime }\rangle ,$$

where Ĥ^*el*^ is the electronic Hamiltonian and the rhsm is obtained from the derivatives of the diabatic energies *V*_ij_ (Sanz-Sanz et al., 2015). *Ab initio* calculations have been performed using the Multi-Reference Configuration-Interaction method, with the aug-cc-pVTZ basis set of Dunning (1989) and Woon and Dunning (1994). The *ab initio* NACMEs are obtained using a first order difference method with an interval of 0.01 *a*_0_, and that for *R*_AB_ = *r* is compared with the fitted one in the bottom panel of Figure 2. The agreement, in form and in position, between both is excellent, indicating that the equation used for the interaction term *V*_12_ is also appropriate to reproduce the NACMEs. The probability density of the first two vibrational states of H_2_(*X*^1^Σ${}_{g}^{+}$) has been included in the top panel of Figure 2, in order to analyze the reason why the reaction is faster when H_2_ is vibrationally excited (Aguillon, 1994, 1998). While the maximum of the probability density of the vibrational state *v*_0_ = 0 is found at 1.4 *a*_0_, those in the first excited vibrational state *v*_0_ = 1 are at 1.25 and 1.73 *a*_0_. The latter is close to the region in which the NACME has is maximum, that explains the enhancement of the reactivity as we shall see in section Reaction Probabilities.

**Figure 3:**
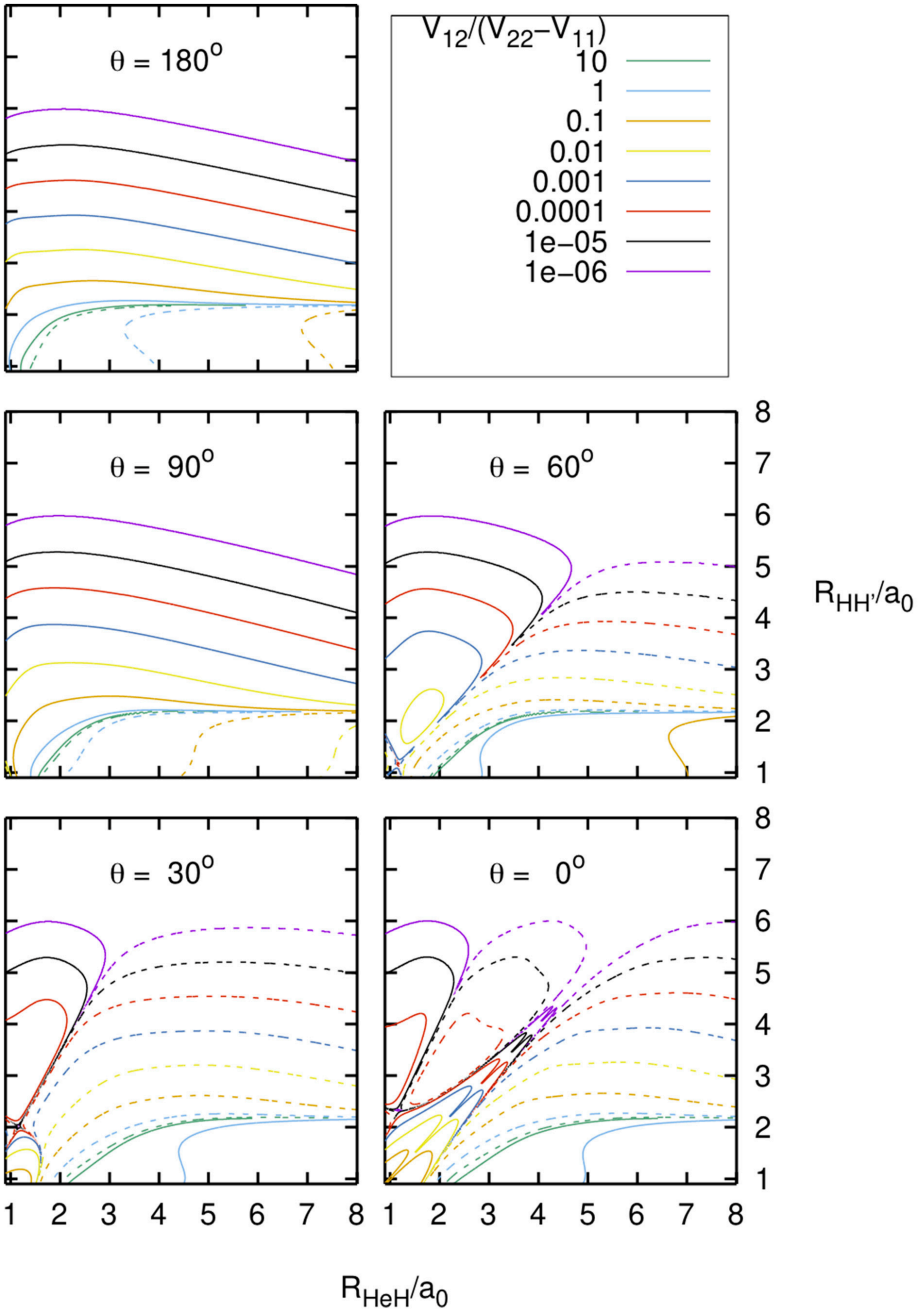
Diabatic PESs: *V*_12_/(*V*_22_-*V*_11_) as function of the nuclear distances *R*_HeH_ and *R*_HH_' = *r*, and of the included angle θ . Solid/dashed curves correspond to positive/negative values.

In Figure 3 the ratio between the interaction term and the energy difference of the diabatic states, *V*_12_/(*V*_22_-*V*_11_), is plotted as a function of the *R*_HeH_ and *R*${}_{\text{H}{\text{H}}^{\text{′}}}$ distances, for several values of the angle θ ≤ 180° between these distances. This ratio is important for the calculation of the adiabatic PESs from the fitted diabatic ones, according to:

(3) $$V_{A/B\;}=\frac{1}{2}\left\{V_{11}+V_{22}-/+\left[{\left(V_{11}-V_{22}\right)}^{2}+4V_{12}^{2}\right]{\;}^{1/2\;}\right\}.$$

As expected, *V*_12_/(*V*_22_-*V*_11_) changes sign and its absolute value is maximum in the region of the diabatic crossing line shown in Figures 2, 3 of Aguado et al. (1993).

In conclusion, these results show that the collisional dynamics of DCT He^+^(^2^*S*)+H_2_(*X*^1^Σ${}_{g}^{+}$)→ He(^1^*S*)+H(^2^*S*)+H^+^ can be investigated with high accuracy in the present diabatic electronic representation, using the *V*_11_, *V*_22_, and *V*_12_ PESs.

### Collision Dynamics

Since many years we are presenting our quantum theory (Petrongolo, 1988) and results of non-adiabatic effects in spectroscopy of triatomics and dynamics of atom+diatom collisions and we here report a brief summary, following our work on the CI dynamics of the OH(*A*^2^Σ^+^)+H(^2^*S*) reaction (Gamallo et al., 2013).

The He^+^+H_2_ collision is described by reactant Jacobi coordinates *R*, *r*, and γ , by a body-fixed reference frame with the *z* axis along ***R***, and by a ${\text{HeH}}_{2}^{+}$ spinless rovibronic Hamiltonian Ĥ, which contains the electronic Ĥ^*el*^ and the total angular momentum $\hat{J}$ operators. Ĥ is represented in an orthonormal basis of diabatic electronic states (1)^2^*A*_1_ and (2)^2^*B*_2_, radial grid |*Rr*>, associated Legendre |*jK*>, and symmetry Wigner states |*K*+*p*>. Here (1)^2^*A*_1_ and (2)^2^*B*_2_ are coupled by Ĥ^*el*^ owing to the CI, ℏ *K* is the Ĵ_*z*_ eigenvalue, and we omit *J* and its space-fixed *Z* component in |*K*+*p*>, where the total parity is $p={\left(-\right)}^{J+K_{\min}}$ with *K*_min_ = 0 or 1. The 2*J*+1 values of *K* are thus factorized in two non-interacting groups, with *K*_min_ ≤ *K* ≤ *J*, of dimensions *J*+1 or *J* according to *K*_min_ = 0 or 1, respectively.

Initial-state-resolved reaction probabilities are computed through the quantum, real WP formalism of Gray and Balint-Kurti (1998) and Meijer et al. (1998), essentially equal to the Chebyshev approach of Guo (2012). Shortly, an *arccos* mapping of the ${\text{HeH}}_{2}^{+}$ time-dependent Schrödinger equation is solved recursively, using a scaled and shifted Hamiltonian Ĥ_s_ and starting from an initial and complex WP |ψ_0_ > = |*a*_0_ > +*i*|*b*_0_ > (Gray and Balint-Kurti, 1998). This initial WP describes the entrance channel He^+^(^2^*S*)+H_2_(*X*^1^Σ${}_{g}^{+}$), with the diabatic electronic state (1)^2^*A*_1_ and the *R*-dependent term

(4) $$\begin{array}{l} g_{0}(R)={\text{π}}^{-1/4}{\text{α}}^{-1/2}exp\left[-\left(R-R_{0}{)}^{2}/2{\text{α}}^{2}\right)\right]\\ \;exp\;{[-i\left(2{\text{μ}}_{\text{R}}E_{0}\right)}^{1/2}\\ \;\left(R-R_{0}\right)],\text{ in atomic units}, \end{array}$$

where μ_R_ is the He^+^+H_2_ reduced mass. The *r* and angular terms of |ψ_0_> are the vibrational |*v*_0_ > and rotational |*j*_0_*K*_0_ > states of H_2_(*X*^1^Σ${}_{g}^{+}$), and finally |*K*_0_+*p*> is the initial overall rotational species. The recursions are

(5) $$\begin{array}{l} |a_{1}\rangle \;={\hat{H}}_{\text{s}}|a_{0}\rangle -{\left(1-{\hat{H}}_{s}^{2}\right)}^{1/2\;}|b_{0}\rangle ,\\ \text{ first complex propagation}, \end{array}$$

(6) $$\begin{array}{l} |a_{\text{n}+\text{2}}\rangle =2{\hat{H}}_{\text{s}}|a_{\text{n}+1}\rangle -|a_{\text{n}}\rangle ,\\ \text{ other real propagations,} \end{array}$$

where the square root in Equation (5) is evaluated with a Chebyshev expansion, and Equation (6) is a standard Chebyshev propagation of just a real WP, which is also absorbed at *R* > *R*_abs_ and *r* > *r*_abs_ by the Gaussians *exp*[–$C_{abs}^{R}$(*R*–*R*_abs_)^2^] and $exp[C_{abs}^{r}{\left(r-r_{\text{abs}}\right)}^{2}]$, respectively. At the end of the propagation, we obtain the probability via a time-to-energy Fourier transform and a flux analysis (Meijer et al., 1998) on the (2)^2^*B*_2_ PES.

We compute non-adiabatic initial-state-resolved reaction probabilities $P_{v_{0}}^{J}$(*E*_coll_), with the initial WP on the ${\text{HeH}}_{2}^{+}$ (1)^2^*A*_1_ diabatic PES and H_2_(*X*^1^Σ${}_{g}^{+}$) in the ground and first excited vibrational state, *v*_0_ = 0 and 1, and in the ground rotational state *j*_0_ = 0. Then the initial *K*_0_ is equal to *K*_min_ = 0 and the total parity *p* is (–)^*J*^. Here we have

(7) $$P_{v_{0}}^{J}\left(E_{coll}\right)={\sum_{v^{\prime }j^{\prime }K^{\prime }}\left|S_{2v^{\prime }j^{\prime }K^{\prime }\leftarrow 1v_{0}00}^{J}\left(E_{coll}\right)\right|}^{2},$$

where (1) and (2) are the diabatic electronic states, *v*′, *j*′, and *K*′ label open vibrational, rotational, and helicity states of the products, respectively, and ***S***^*J*^ is the state-to-state parity adapted *S*-matrix at *J*. The total ICS is then defined by

(8) $$\sigma_{v_{0}}\left(E_{coll}\right)=\frac{\pi }{2{\text{μ}}_{R}E_{coll}}\sum_{J}\left(2J+1\right)P_{v_{0}}^{J}\left(E_{coll}\right),$$

and the initial-state-resolved rate constant is

(9) $$k_{v_{0}}\left(T\right)={\left(\frac{8}{\pi {\text{μ}}_{R}k_{B}^{3}T^{3}}\right)}^{1/2}\int_{0}^{\infty }E_{coll}\sigma_{v_{0}}\exp\left(-E_{coll}/k_{B}T\right)dE_{coll},$$

where *T* is the temperature and *k*_B_ is the Boltzmann constant.

The calculations are done up to *J* = 150, using the coupled-channel formalism with Coriolis couplings among the *K* values, up to *K*_max_, that are necessary for converging the probabilities. Considering the H–H permutation symmetry, the numerical parameters of the WP propagations are listed in Table 1: they correspond to 7,053,300 basis states at *K* = 0, including two coupled electronic states. These parameters span the whole range of *E*_coll_ from 0.0002 to 3 eV, with Δ*E*_coll_ = 0.0001 or 0.001 eV below or above 0.2 eV, respectively. The most important parameters that affect the convergence of the calculations are the number of the propagation kilo-steps, *kstep*, the maximum *K* value, *K*_max_, and the dimension of the *R* grid, *nR*. We shall see in the next sections some convergence results with respect to *kstep* and *K*_max_. The probabilities obtained with *nR* = 461 of Table 1 are practically indistinguishable by those corresponding to *nR* = 559. All calculations are done with our *J-K*-parallelized Open MPI codes.

**Table 1:** Parameters of the quantum dynamics calculations.

Initial Gaussian *g*_0_(*R*), Equation (4), α, *R*_0_, and *E*_0_	0.2, 20, and 1.5 eV
*R* range and number of grid points	0.5–45 and 461
*r* range and number of grid points	0.5–15 and 153
Number of (associated) Legendre functions |*j*0>	50 (*j* = *even*)
*R* and *r* absorption start at	30 and 11
*R* and *r* absorption strength	0.0005 and 0.005
Flux analysis at *r*	10

*Values in atomic units, unless otherwise specified*.

## Collision Results

### Reaction Probabilities

**Figure 4:**
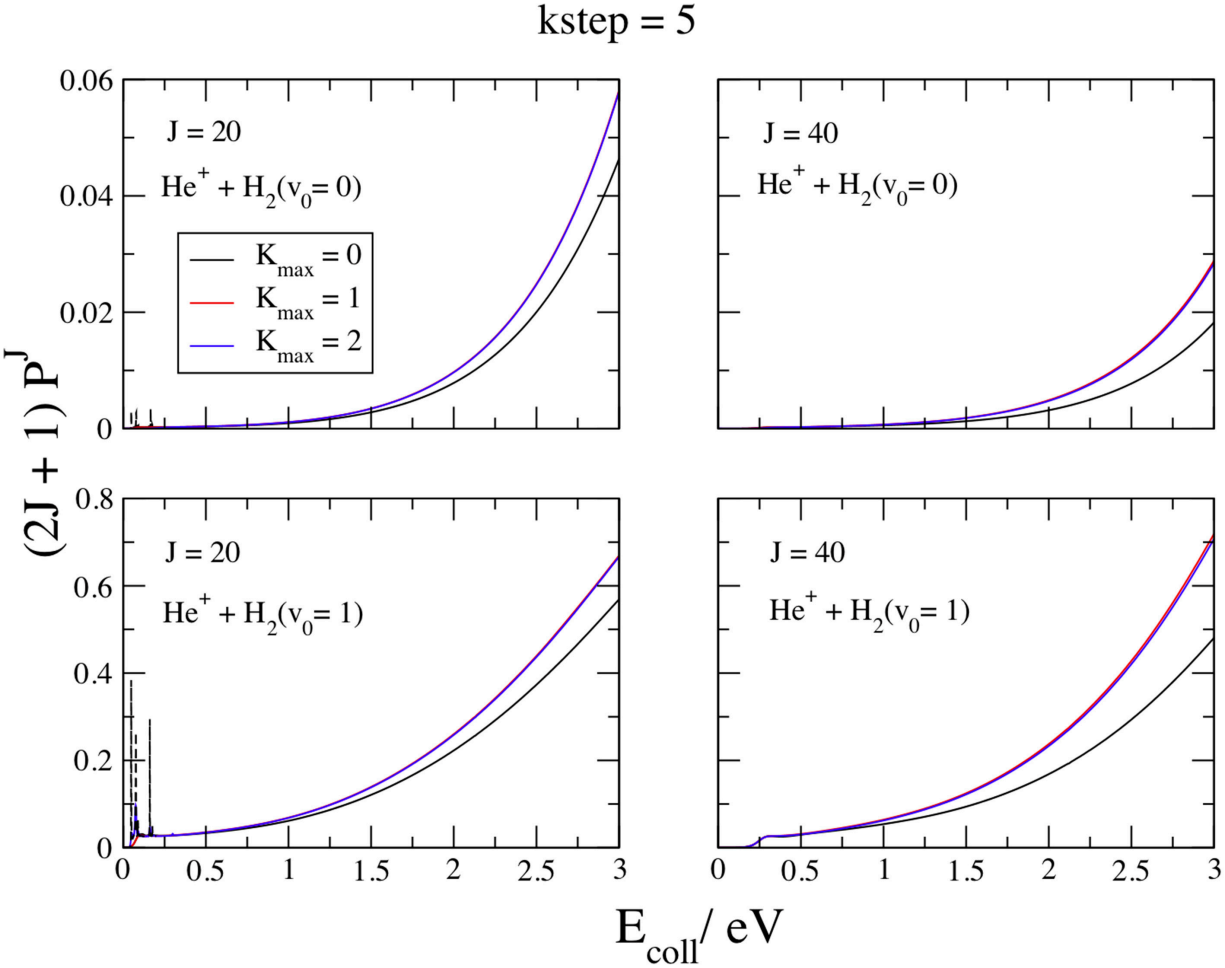
*v*_0_ = 0, 1; *J* = 20, 40; *kstep* = 5. Convergence of the opacities (2*J*+1)$P_{v_{0}}^{J}$ with respect to *K*_max_.

We plot in Figure 4 the opacities functions (2*J*+1)$P_{v_{0}}^{J}$(*E*_coll_) at *J* = 20 and 40, *v*_0_ = 0 and 1, *E*_coll_ ≤ 3 eV, and *K*_max_ = 0, 1, and 2, with *kstep* = 5, which converges the probabilities within 0.1% above ~0.5 eV. The curves at *v*_0_ = 0 and 1 present a similar behavior, namely narrow resonance features at *J* = 20 and *E*_coll_ ≤ 0.5 eV and a smooth, fast increase above. From the comparison among lower and upper panels we see that *v*_0_ = 1 is more reactive than *v*_0_ = 0 by ~one order of magnitude. Of course, this finding reflects the ${\text{HeH}}_{2}^{+}$(Ã^2^*A*′) early potential barrier of 1.34 eV and the *v*_0_ = 1 density maximum at 1.73 *a*_0_ (Figure 2), close to the NACME maximum. Comparing the curves inside each panel, we observe a very fast convergence in *K*_max_, so that the Coupled-State approximation (McGuire and Kouri, 1974; Pack, 1974) at *K*_max_ = 0 gives already reasonable results. Just two *K*s converge the plots within the graphical accuracy, implying that the Coriolis couplings between *K* and *K* ± 1 vanish quickly when the initial *K*_0_ is equal to zero. To reach the high accuracy claimed above at all the partial waves required to give convergent ICSs in all the collision energy range considered, we employ *K*_max_ = 2 and 3 for *v*_0_ = 0 and 1, respectively. In the *J* = 20 left panels, the dashed lines show the convergent results below 0.5 eV and the weight of the resonances with respect to the background. The resonance features are probably due to rotational metastable states of the *C*_2v_ ion-induced dipole minimum in the entrance channel. These states, embedded in the collisional continuum, are particular Feshbach resonances induced by the CI (Cederbaum and Friedman, 2003), with lifetimes of the order of the nano-second. The *v*_0_ = 0 opacities then increase in a monotonic way above 0.6 eV up to a maximum value of ~0.06 at 3 eV, that is one-two orders of magnitude larger than the strongest resonance. The *v*_0_ = 1 opacities show the same behavior, but the value at 3 eV is just two times larger than the resonance maximum. However, to converge the resonance energy pattern very different values of the convergent parameters *K*_max_ and *kstep* are required.

**Figure 5:**
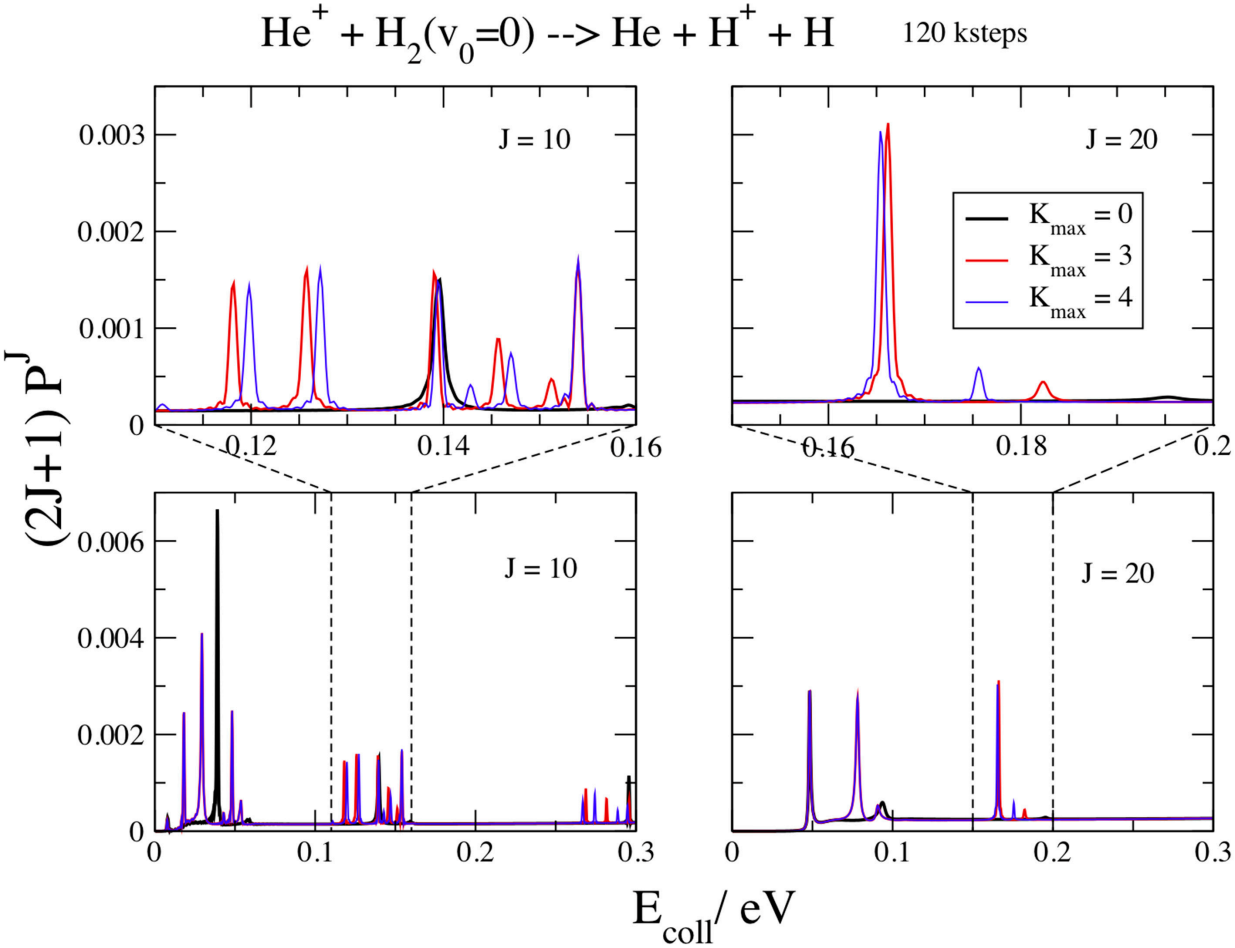
*v*_0_ = 0; *J* = 10, 20; *kstep* = 120. Convergence of the opacities (2*J*+1)$P_{v_{0}}^{J}$ with respect to *K*_max_.

We show in Figure 5 the opacities at *J* = 10 and 20, for *v*_0_ = 0, *E*_coll_ ≤ 0.3 V, *K*_max_ = 0, 3, and 4, and *kstep* = 120. In the upper panels a blow up of 0.05 eV also shows the details of the resonance features. From this figure we can observe that the convergence in *K*_max_ is slower than for high energies. The curves at *K*_max_ = 0 exhibit less peaks and the resonances shift in energy by increasing this parameter. Three main groups of resonances could be identified, decreasing and then disappearing at high *E*_coll_. This result suggests that the different peaks inside each group correspond to different bending energies of the collision complex (Aquilanti et al., 2005) while different groups correspond to different energies of the symmetric stretching motion. The *v*_0_ = 1 curves have a similar behavior, with the *K*_max_ convergence slightly faster and the resonances at lower energies.

**Figure 6:**
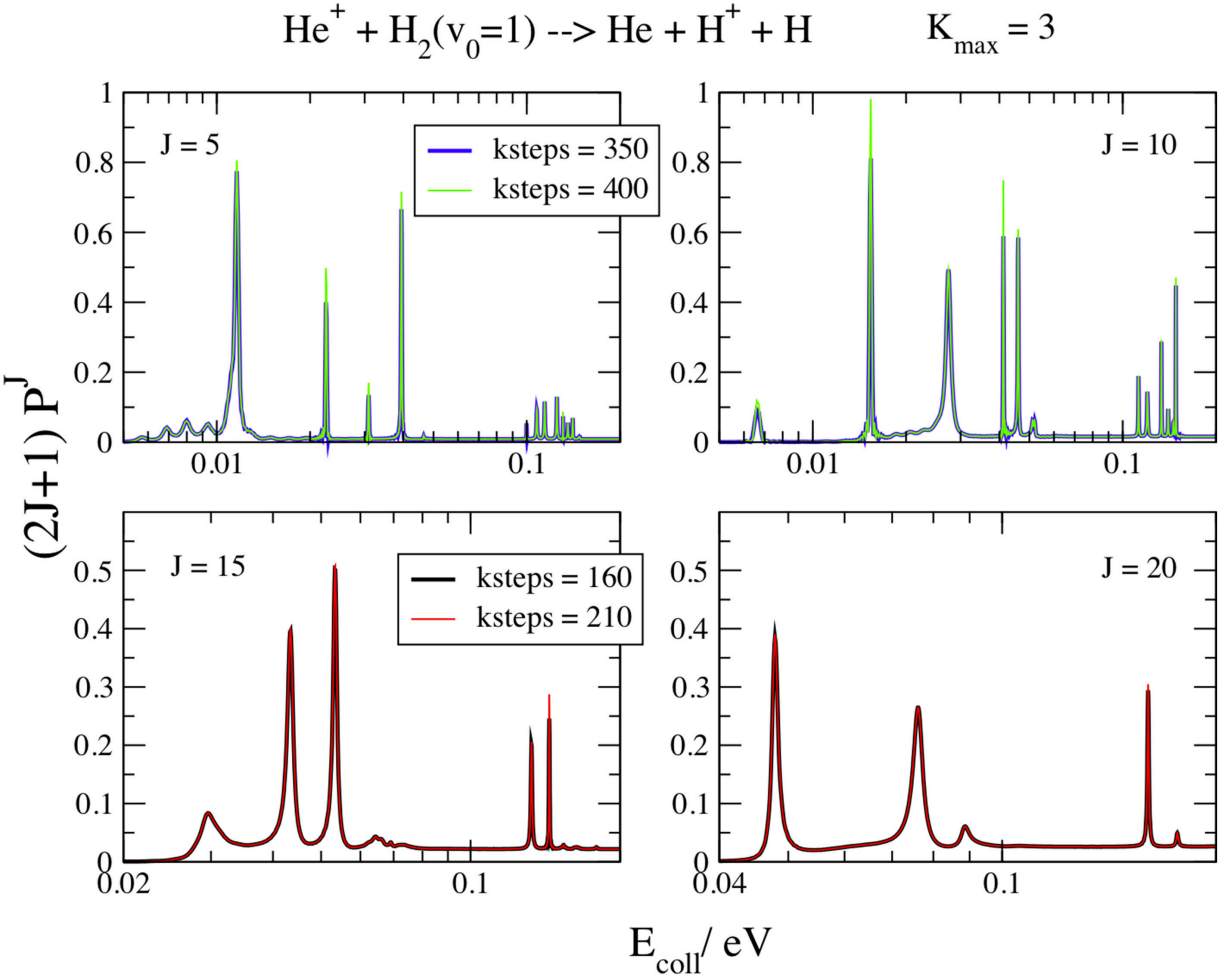
*v*_0_ = 1; *J* = 5, 10, 15, 20; *K*_max_ = 3. Convergence of the opacities (2*J*+1)$P_{v_{0}}^{J}$ with respect to *kstep*.

In Figure 6 the resonance energy patterns at *J* = 5, 10, 15, and 20, *v*_0_ = 1, and *K*_max_ = 3 are plotted for *E*_coll_ ≤ 0.11 eV at different *kstep* values. The *K*_max_ value gives a graphical convergence to the curves and the logarithmic scale of the energy points out the resonance details. Here *kstep* is much greater than for the background and changes markedly with *J* and *E*_coll_. In fact, *kstep* = 350 is still not enough to converge all the *J* = 5 and 10 features, especially below 0.01 eV, but *kstep* = 160 perfectly converges the resonances at *J* = 15 and 20. As expected, the slowest convergent resonances are the narrowest ones, with largest lifetimes (Aquilanti et al., 2004). Moreover, the lifetimes of the collision complexes decrease at high *J* and *E*_coll_, because the centrifugal barrier increases and the resonances are less trapped inside the shallower well. Above *J* = 25 the well is so shallow that it does not support any resonance and the features disappear. Of course, increasing *kstep* means increase the collision time that must be of the same order of magnitude of the lifetime of the resonance intermediate to give convergent results.

### Integral Cross Sections and Rate Constants

**Figure 7:**
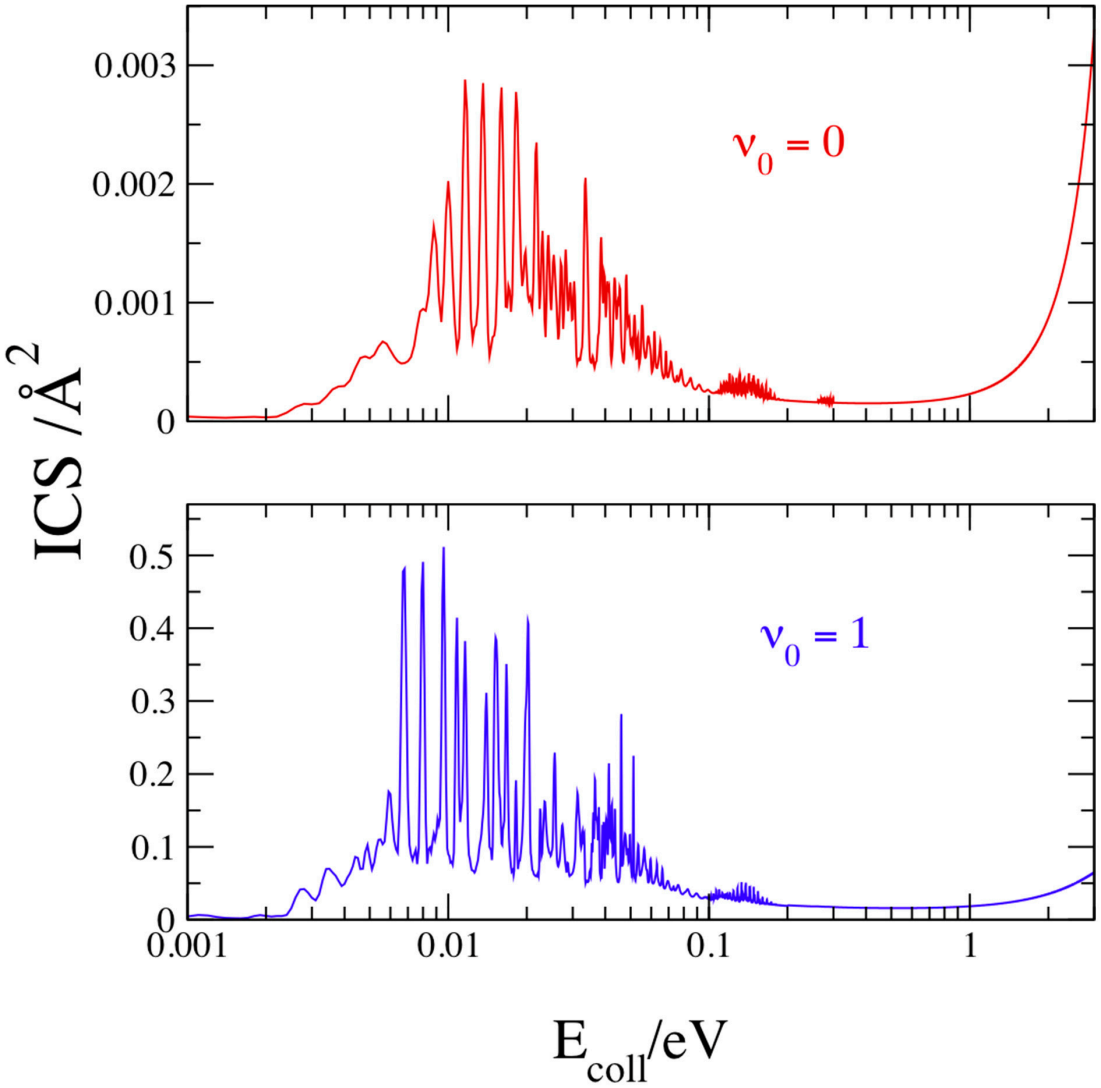
Initial-state-resolved ICSs at *v*_0_ = 0 (red) and 1 (blue).

We plot in Figure 7 the total ICS at *v*_0_ = 0 and 1, with *J* ≤ 150 to obtain convergent results up to 3 eV, noting that the convergent requirements change markedly with *E*_coll_ and *J*. To minimize the computational effort, the total number of partial waves was therefore shared in different groups, and different values of *K*_max_ and *kstep* were employed in each group, as shown in Table 2.

**Table 2:** Parameters of ICS calculations.

***v*_**0**_**	***J***	***kstep***	***K*_**max**_**
0	0–24	160	4
	25–52	40	3
	53–82	20	3
	83–150	10	1
1	0–14	350	3
	15–25	160	3
	26–38	110	3
	39–102	20	2
	103–150	10	1

Notwithstanding the large *kstep* used for the lowest partial waves, test calculations show that some narrow ICS peak slightly increases with more steps. With these input data, a convergence within 0.1% is reached only by the resonance peaks above 0.05 eV. The results in Figure 7 confirm the general scenario of the opacities: sharp resonances below 0.3 eV and a monotonic increase at larger *E*_coll_. The *J* sum now merges the resonances in two main groups, the first and stronger below 0.1 eV, and the latter and weaker from 0.1 to 0.3 eV. At *v*_0_ = 0, the strongest resonance is equal to 0.003 Å^2^ at 0.0116 eV and the maximum at 3 eV has nearly the same value. At *v*_0_ = 1, the strongest resonance is larger than the value at 3 eV by ~one order of magnitude, and is equal to 0.455 Å^2^ at 0.0096 eV. In conclusion, the one-quantum vibrational excitation of H_2_(X^1^Σ${}_{g}^{+}$) increases the cross section by more than two orders of magnitude at ~0.01–0.02 eV and by ~20 times at 3 eV.

As said in the Introduction, only the semiclassical theoretical works by Aguillon (1994, 1998) have obtained ICSs at or below 3 eV. We present in Table 3 a comparison between our quantum ICSs and those estimated from Figure 4 of Aguillon (1998), showing the good agreement between the results that differ at the most by ~24% at *v*_0_ = 0 and *E*_coll_ = 2 eV. Taking into account the different theoretical treatment and the small reactivity at these conditions, this implies that the Aguillon semiclassical treatment of the DCT reaction works remarkably well at this collision energies, where the ICSs do not present any quantum resonance.

**Table 3:** Present quantum ICSs/Å^2^ vs. those semiclassical (Aguillon, 1998).

***v*_**0**_**	***E*_**coll**_/eV**	**present work**	**(Aguillon, 1998)**
0	2	8.7 × 10^−4^	(~7 × 10^−4^)
	3	3.3 × 10^−3^	(~3 × 10^−3^)
1	2	3.5 × 10^−2^	(~3.5 × 10^−2^)
	3	6.5 × 10^−2^	(~6 × 10^−2^)

**Figure 8:**
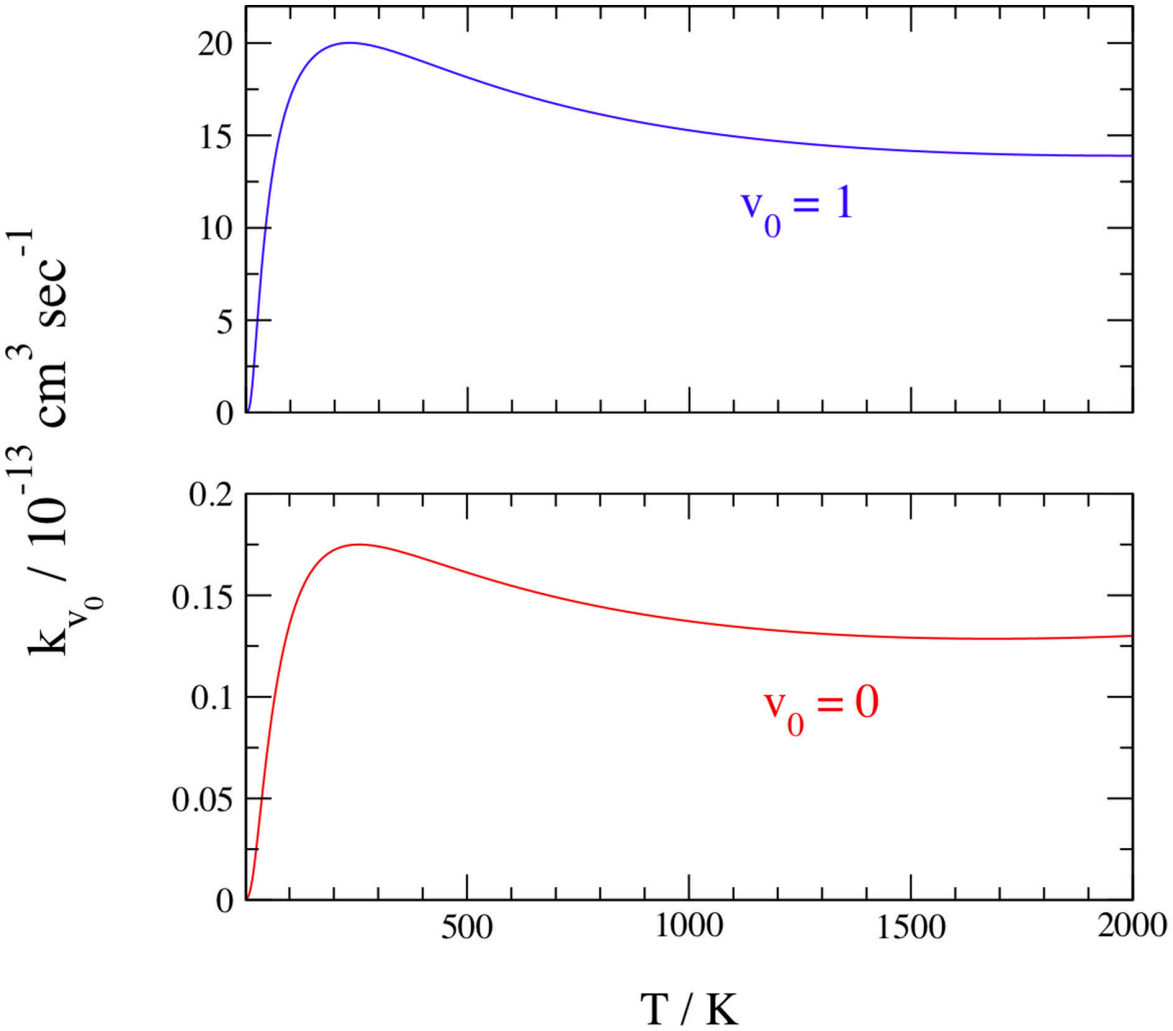
Initial-state-resolved rate constants at *v*_0_ = 0 (red) and 1 (blue).

Finally, we report in Table 4 and Figure 8 the initial-state-resolved rate constants *k*_*v*_0__(*T*), at *v*_0_ = 0 and 1, as functions of the temperature *T* up to 2,000 K. Their accuracy is ~2%, considering the slow convergence of the resonances, and the rates are stable with respect to changes of the parameters in Tables 1, 2. Both rates increase quickly by a factor of ~30 from 20 to 200 K, with a maximum at ~250 K equal to 1.75 10^−14^ and 1.97 10^−12^ cm^3^ sec^−1^ for *v*_0_ = 0 and 1, respectively. This behavior is associated with the sharp ICS resonances above ~0.006 eV and is similar to that of H+HeH^+^ (De Fazio, 2014). Note that a rate-constant maximum was also found in the adiabatic ground PES dynamics of H+HeH^+^ (Esposito et al., 2015) using the PES of Ramachandran et al. (2009), but it was at ~10,000 K and due to a complete different mechanism. Then the rates slowly decrease with *T* and become nearly constant above 1,500 K, where they differ by two orders of magnitude. On the overall, this behavior is due to the ICS resonances shown in Figure 7 overlapped to a nearly Langevin decrease of the associated cross sections up to ~0.5 eV.

**Table 4:** Rate constants *k*_0_ and *k*_1_/cm^3^ s^−1^.

***T*/K**	***k*_**0**_/10^**−14**^**	***k*_**1**_/10^**−12**^**
50	0.73	1.12
100	1.36	1.71
200	1.72	1.99
300	1.74	1.98
500	1.61	1.81
1,000	1.37	1.53
2,000	1.30	1.39

Because we have considered just the ground rotational state of H_2_(*X*^1^Σ${}_{g}^{+}$), *j*_0_ = 0, the present rate constant at *v*_0_ = 0 and 300 K underestimates by a factor of ~6 the thermal experimental *k*(330) = (1.1 ± 0.1) × 10^−13^cm^3^s^−1^ (Johnsen et al., 1980), which is however 39 years old. The agreement increases if we consider H_2_ in the excited rotational states that are open at 300 K, because test calculations suggest that the reactivity can be enhanced by ~33% when *j*_0_ = 1. Taking into account the H_2_(*X*^1^Σ${}_{g}^{+}$) Fermi-Dirac nuclear spin statistics, the room-temperature populations of *j*_0_ = 0 and 1 are ~0.13 and 0.66, respectively, and this implies a rotational enhancement of the rate constant by a factor of ~7. Owing to the room-temperature branching ratio of *para*- and *ortho*-H_2_ and the rotational effects on the reactivity, we can roughly estimate than *ortho*-H_2_ is ~4 times more reactive than the *para* species.

**Figure 9:**
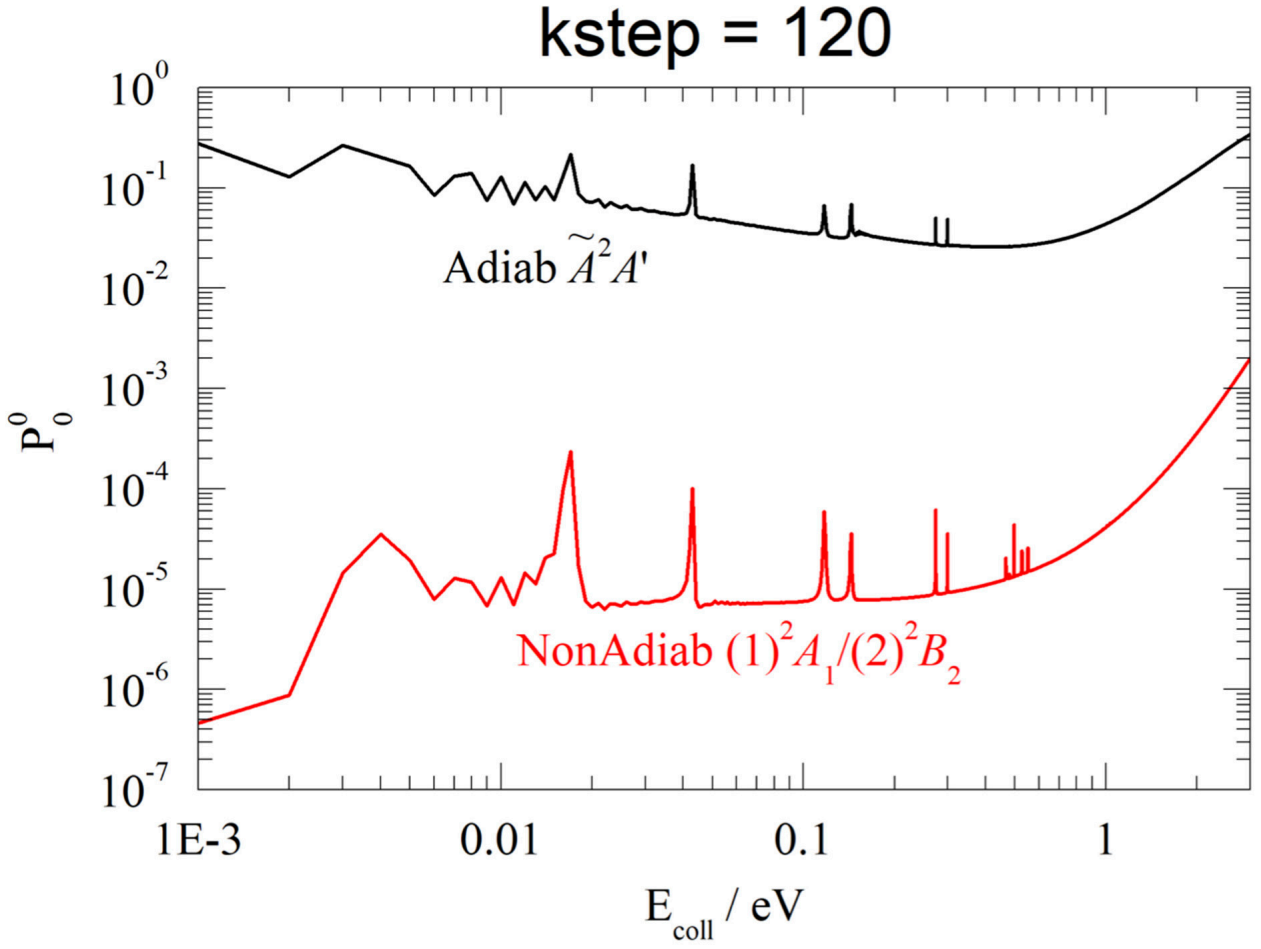
*v*_0_ = 0, *J* = 0, *kstep* = 120. Adiabatic Ã^2^*A*' (black) and non-adiabatic (1)*A*_1_/(2)*B*_2_ (red) probabilities.

In closing this section, we have also done some test calculations of the reaction probabilities in the adiabatic approximation, on the Ã^2^*A*' PES of ${\text{HeH}}_{2}^{+}$ (see Figure 1). Without reaching the accuracy and the stability of the results in section Reaction Probabilities, we present an example at *v*_0_ = 0, *J* = 0, and *kstep* = 120, contrasting in Figure 9 adiabatic Ã^2^*A*' and non-adiabatic CI (1)^2^*A*_1_/(2)^2^*B*_2_ results. Although both probabilities present a resonance structure at low *E*_coll_ and increase above ~1 eV, the reactivity is dramatically different, with the Ã^2^*A*′ probability hugely larger than the CI one, from 6 orders of magnitude at 0.001 eV to 2 orders at 3 eV. This finding shows that the Ã^2^*A*′ adiabatic PES drives the WP into the exothermic product channel, following a *C*_*s*_ pathway that avoids the *C*_2v_ barrier of the CI. On the contrary, the non-adiabatic CI WP remains essentially on the initial and repulsive (1)^2^*A*_1_ diabatic PES *V*_11_, owing to the weak non-adiabatic interaction. As expected, only at energies larger than 3 eV the two probabilities seem to merge and the adiabatic approximation is probably less worse. We roughly estimate that the adiabatic Ã^2^*A*′ rate constants at 330 K is ~1,000 times larger than the experimental value (Johnsen et al., 1980).

## Conclusions

In this article we have presented quantum non-adiabatic DCT results of the He^+^+H_2_ collision, employing an electronic diabatic representation, previously computed by Aguado et al. (1993), a WP time dependent formalism, and a flux analysis. Specifically, we have taken into account the non-adiabatic CI coupling between the first two excited diabatic PESs of ${\text{HeH}}_{2}^{+}$. Dynamical calculations are performed for the ground and first excited vibrational states of H_2_, for investigating vibrational effects on the DCT dynamics, and collision energies up to 3 eV are considered. Reaction probabilities and ICSs present strong and narrow resonance features up to 0.5 eV, due to quasi-bound molecular states embedded in the continuum and trapped in the ion-induced dipole minimum of the reactant channel, near the CI. These features are very hard to converge and affect all the DCT dynamics. These intense resonance features have a determinant role in the radiative charge-transfer formation (Mrugala et al., 2013) of stable ${\text{HeH}}_{2}^{+}$, probably present in the interstellar medium (Tennyson and Miller, 1987). At higher collision energies the computational load reduces drastically owing to a near conservation of the helicity quantum number. Our results are in reasonable agreement with previous experimental and theoretical studies of this reaction, confirming the strong vibrational enhancement previously founded. In fact, rate constants increase of about two orders of magnitude just adding one vibrational quantum to the H_2_ reactants.

At the best of our knowledge, these are the first rigorous quantum DCT calculations presented for a chemical system, made possible by the joint implementation of time-dependent WP, time-to-energy Fourier transform, and flux methods. On the other hand, a more rigorous approach, as time independent close coupling calculations, cannot be attempted at the present state-of-the-art of the reaction dynamics theories, because of the difficulty of these methods to treat the three-bodies breakup (see e.g., (Pack et al., 1998), and references therein). The results achieved could have relevant consequences in astrophysics and in particular in the early Universe evolution models (Bovino et al., 2011). Although the reaction is probably negligible when the molecular hydrogen is relaxed in its ground vibrational state, the strong increase with the vibrational energy suggests that it could have a role during the early stages of the adiabatic expansion at high redshift, destroying the molecular hydrogen formed and slowing down further cooling of the primordial gas. Of course, to determinate its role, evolution Universe models with at least a vibrational resolution of the chemical network (Coppola et al., 2011, 2012) are required. Moreover, this reaction could be also relevant in other different astrophysical environments, as for example in the study of the Sun atmosphere where the temperature is very high and hydrogen and helium are very abundant species.

## Author Contributions

All authors listed have made a substantial, direct and intellectual contribution to the work, and approved it for publication.

### Conflict of Interest Statement

The authors declare that the research was conducted in the absence of any commercial or financial relationships that could be construed as a potential conflict of interest.
